# HIV-associated adipose redistribution syndrome (HARS): etiology and pathophysiological mechanisms

**DOI:** 10.1186/1742-6405-4-14

**Published:** 2007-06-27

**Authors:** Kenneth Lichtenstein, Ashok Balasubramanyam, Rajagopal Sekhar, Eric Freedland

**Affiliations:** 1 University of Colorado Infectious Disease Group Practice, Denver, CO, USA; 2 Division of Diabetes, Endocrinology & Metabolism, Baylor College of Medicine, Houston, TX, USA; 3 EMD Serono, Inc., Rockland, MA, USA

## Abstract

Human immunodeficiency virus (HIV)-associated adipose redistribution syndrome (HARS) is a fat accumulation disorder characterized by increases in visceral adipose tissue. Patients with HARS may also present with excess truncal fat and accumulation of dorsocervical fat ("buffalo hump"). The pathophysiology of HARS appears multifactorial and is not fully understood at present. Key pathophysiological influences include adipocyte dysfunction and an excessive free fatty acid release by adipocyte lipolysis. The contributory roles of free fatty acids, cytokines, hormones including cortisol, insulin and the growth hormone-adipocyte axis are significant. Other potential humoral, paracrine, endocrine, and neural influences are also discussed.

## Background

Active antiretroviral therapy has reduced mortality of AIDS (acquired immune deficiency syndrome) and increased both the quality of life and longevity in patients infected with HIV (human immunodeficiency virus). However, long-term effects of HIV infection are increasingly observed [1,2]. Among the more apparent effects are changes in fat distribution or "lipodystrophy," which includes fat loss or "lipoatrophy" and/or an increase in fat accumulation, also known as "lipohypertrophy" [3]. The pathological aberrations of fat metabolism seen in some HIV-infected patients remain inadequately understood and controversial despite extensive studies[4]. These changes may be an outcome of HIV itself or that of active antiretroviral therapy. Lipoatrophy and lipohypertrophy may occur separately or together in an individual.

The term HIV-associated adipose redistribution syndrome (HARS) describes a fat accumulation disorder in patients with HIV, which has specific characteristics. The primary characteristic is an increase in the amount of visceral adipose tissue (VAT) [5], often observed in patients while they are undergoing active anti-retroviral therapy[6]. In addition, adipose tissue may accumulate subcutaneously in regions such as the trunk and the dorsocervical area – where a significant depot may be referred to as a "buffalo hump". Further, metabolic perturbations including insulin resistance, glucose intolerance, dyslipidemia and hypertension, as well as body image distress may accompany these changes. These metabolic disturbances may portend the development of other, more serious, medical conditions including diabetes mellitus and cardiovascular disease [7-9]. While the most commonly observed form of HIV lipodystrophy is lipoatrophy[10], patients with HARS may or may not present with lipoatrophy of subcutaneous adipose tissue (SAT), including abdominal SAT[11]. In this review, we address potential pathophysiologic mechanisms to explain HARS and its associated health risks.

## Etiology and Pathogenesis of HARS

Although the underlying mechanisms of HIV-associated lipodystrophy and HARS remain unclear, investigators show increasing interest in teasing apart the various aspects of HIV-lipodystrophy. While peripheral fat loss and central fat accumulation may be seen in the same patient, there is no statistically significant relationship between the two conditions[12], so a single metabolic process is unlikely to simultaneously decrease and increase fat in the same patient. Though the term "redistribution" is used in HARS and the Fat Redistribution and Metabolic Changes in HIV Infection Study (FRAM), it is not meant to imply the migration of fat from one depot to another. Rather, it refers to the overall re- or maldistribution of fat as adipose tissue atrophies in some areas, e.g., face, buttocks and limbs, and at the same time fat accumulates or hypertrophies in other areas, e.g. in the abdomen as VAT.

The etiology of HIV lipodystrophy, and HARS in particular, is certainly multifactorial. Intrinsic host factors, disease status, treatment duration and type, as well as other factors probably all play a role. Evidence summarized by Lichtenstein[13] suggests that significant risk factors for fat loss include exposure to and duration of thymidine analogues (particularly the older ones, stavudine, zidovudine), age, CD4-positive count, viral load, duration of therapy, and white race. In contrast, risk factors for abnormal fat accumulation include duration of therapy, CD4-positive count, viral load, age, protease inhibitor use (particularly the older ones, indinavir, nelfinavir), and female sex[14]. These differences in risk factors for the two manifestations of lipodystrophy suggest that the pathogenic mechanisms for fat redistribution, loss versus gain, are probably independent and the result of complex interactions of host, disease and drug factors.

## Pathogenic Influences

Visceral and subcutaneous fat depots are genetically and metabolically different from each other. Surgical removal of VAT in animals and humans improved insulin resistance and glucose while removal of SAT did not [15-17]. Acute increases in glucose uptake into adipose tissue by either hyperglycemia or hyperinsulinemia increased the expression of several fat-derived peptides to a greater extent in VAT than in SAT[18].

It is known that the metabolic function of adipose tissue is controlled by numerous humoral, paracrine, and intracrine factors[19], as well as by sympathetic innervation (stimulating lipolysis) and parasympathetic innervation (parasympathectectomy can induce insulin resistance in the denervated fat depot) [20,21]. It is likely that disruption of these complex interacting regulatory pathways, in various ways (e.g. by HIV itself or its treatment), accounts for at least some of the fat maldistribution and metabolic consequences seen in HARS. Chronic disruption of paracrine interactions between lymphoid and adipose tissue may play a role in HARS[19], while HIV or antiretroviral agents may adversely affect neural pathways to different fat depots[20].

## Adipocyte Dysfunction

Among the observed endocrine and cytokine alterations in HIV lipodystrophy are increased levels of insulin[22] and tumor necrosis factor-α (TNF-α)[19] and defects in the secretion of growth hormone (GH), all of which may alter adipocyte function. The problem in understanding the pathogenesis of HARS, however, is that the observed metabolic effects associated with HIV lipodystrophy are the sum of multiple metabolic disturbances, each a separate component.

Disruption of specific regulatory controls of VAT may account for HARS. VAT may differ from SAT tissue in function, however, recent data from FRAM suggests that both VAT and upper trunk SAT are independently associated with insulin resistance[23]. A critical VAT threshold may exist, whereby signs of metabolic syndrome do not appear until the VAT mass exceeds this level for a given individual[24].

### Leptin

Leptin is an adipocyte-derived hormone and its plasma levels directly correlate with adipose tissue mass (SAT rather than VAT). Leptin is involved in the regulation of energy homeostasis and has peripheral effects on fatty acid oxidation. Fasting leptin levels have been shown to correlate with total body fat concentrations in HIV-infected patients[25]. Nagy et al. found that leptin levels were lowest in HIV patients exhibiting lipoatrophy, intermediate in those with mixed lipodystrophy or normal body habitus, and highest in those with lipohypertrophy[26]. These findings could be attributed to a reduction in leptin synthesis in those with lipoatrophy and reduced SAT[27] and excess circulating levels of leptin due to leptin resistance in those with VAT hypertrophy[25]. This leptin resistant state might also be associated with metabolic syndrome and insulin resistance in HIV patients with lipohypertrophy[25].

### Adiponectin

Adiponectin improves insulin sensitivity, and reduced adiponectin is associated with insulin resistance, hypertriglyceridemia, and fat redistribution in HIV patients treated with HAART[28]. An inverse relation has been found between adiponectin levels and VAT mass, serum triglycerides and insulin resistance in HIV patients. These findings are consistent with the role of VAT in the development of insulin resistance and lipid abnormalities associated with the metabolic syndrome. Since adiponectin expression is higher in SAT than VAT, accumulation of VAT combined with SAT loss may lead to decreased adiponectin production in both lipoatrophy and HARS[25].

Hadigan et al. demonstrated depot-specific regulation of glucose uptake and insulin sensitivity in fat and muscle, with a possible role of adiponectin associated with area of VAT and total body disposal of glucose[29]. Although glucose uptake by SAT was greater in HIV-infected than non-infected men, there was no difference in glucose uptake by VAT. VAT area was strongly correlated (r^2 ^= 0.94, P < 0.0001) with whole-body glucose disposal, perhaps mediated by adiponectin. Lundgren et al. have demonstrated that VAT of non-HIV patients normally has a two-fold higher glucose uptake rate compared with SAT[30].

### Plasminogen Activator Inhibitor Type-1

Proinflammatory cytokine activity may be increased in HIV-infected patients with lipodystrophy[31]. Plasminogen activator inhibitor type-1 (PAI-1) is a multifaceted proteolytic factor. It plays an important role in signal transduction, cell adherence and cell migration; and is found in plasma and adipose tissue, is increased in obesity, and predicts cardiovascular disease. PAI-1 is associated with proinflammatory adipokines, such as TNF-α and interleukin-6 (IL-6). It has also been reported to be elevated in HIV-associated lipodystrophy syndrome, along with a three-fold elevation of visceral fat, and reduced peripheral fat[32].

### Uncoupling Proteins

The central role of uncoupling proteins in the several pathways that control VAT function may explain the multifactorial nature of HARS metabolic complications[33]. Recent results establish uncoupling protein-2 as a key component of β-cell glucose sensing, and as a critical link between obesity, β-cell dysfunction and type 2 diabetes[34].

### Local Cortisol and 11-β-HSD1

Although elevated cortisol or abnormalities of the glucocorticoid receptor have been excluded as a potential cause of HIV lipodystrophy, this does not rule out the possibility of a local effect of cortisol on adipose depots in HIV lipodystrophy[35]. Cortisol is known to promote adipogenesis[13], thus increased local cortisol concentrations may increase VAT. VAT cells express high levels of the enzyme 11-beta hydroxysteroid dehydrogenase type 1 (11-β-HSD1), which helps catalyze inactive cortisone to cortisol. Sutinen et al. showed significantly higher levels of 11-β-HSD1 in the SAT of HIV lipodystrophy patients compared to non-lipodystrophy cases[36]. Transgenic mice overexpressing this enzyme develop a full-blown metabolic syndrome with obesity and increased amounts of VAT [37]. Insulin can stimulate 11-β-HSD1 [38], and insulin resistance could lead to elevated insulin levels, which might ultimately increase VAT by stimulating the increase in local cortisol production.

### Growth Hormone and 11-β-HSD1

Evidence suggests that reduced growth hormone (GH) may play a role in the local regulation of cortisol in VAT by 11-β-HSD1. GH inhibits 11-β-HSD1 in adipose tissue, which may result in reduced active cortisol to stimulate VAT production[22]. D'Amico and colleagues note decreased lipolytic rates with a physiological replacement dose of GH administered to HARS patients with GH deficiency identified by dynamic testing[39]. They suggest that this effect may be modulated by a reduction in adipose cortisol concentrations[39].

### Cytokines, Free Fatty Acids and Insulin Resistance

Long-term adaptive changes occur in adipose tissue in chronic disease[19]. Visceral fat drains via the portal vein into the liver and may contribute to elevated serum triglycerides and insulin resistance seen in lipodystrophy patients. It is possible that increased cytokine (TNF-α, IL-6) secretion from adipose tissue, and increased systemic proinflammatory cytokine activity may play a role in the remodeling of adipose tissue and metabolic abnormalities seen in patients with HIV-associated lipodystrophy [31]. The increase in the plasma levels of free fatty acids (FFAs), TNF-α and IL-6, along with a decrease in adiponectin, together could induce liver and skeletal muscle insulin resistance and dyslipidemia. The resulting increased disposal of FFA would be preferentially stored in VAT, leading to VAT lipohypertrophy [40]. Therefore, visceral adiposity may be an initial adaptive response of the body to prevent a rise in levels of FFAs, and an effort to reduce potential lipotoxic damage to other organs resulting from the increase in total triglycerides, triglyceride-rich lipoproteins and raised low-density lipoprotein levels.

## Effects of Lipolysis

In the fasted state, HARS is characterized by an excessive FFA release by adipocyte lipolysis, resulting in a greater net delivery of FFA to the plasma, ultimately resulting in greater hepatic delivery and export as triglyceride-rich very low density lipoprotein (VLDL) leading to hypertriglyceridemia[22,41,42]. In the fed state, a different mechanism results in a second contribution to the hypertriglyceridemia associated with HARS. Examination of the disposal of labeled triglyceride from the plasma chylomicron pool in HARS patients showed a marked retardation of the labeled fatty acids from the chylomicrons compared to controls. Further, of the small amount cleared, a greater proportion of the released FFA was found within the plasma rather than taken into the adipocyte in HARS patients suggesting that there was an adipocyte defect resulting in impaired fat storage [43].

The mechanism underlying lipodystrophy may involve the regulation of adipocyte hormone-sensitive lipase; the result is an accelerated rate of whole-body lipolysis that facilitates the "redistribution" of fat. Results of a study in men with both peripheral fat loss and central fat accumulation, as well as dyslipidemia suggested that a regulatory defect in adipocyte lipolysis could account for both the dyslipidemia and the peripheral fat loss [42]. Hyperlipolytic activity and increased release of FFAs would promote processes for which fatty acids are substrates, for example, the hepatic extraction and conversion to glycerolipids. This mechanism could explain the hypertriglyceridemia seen in HIV-infected patients with lipodystrophy, but does not explain the central adiposity seen in some of them. The authors conjecture that either decreased lipolysis or increased deposition of fatty acids outstripping lipolysis in visceral fat depots might account for the observed central adiposity [42]. This is further discussed below.

Sekhar and colleagues have posited a basic defect in fatty acid metabolism in peripheral adipocytes in HARS patients to account for: the acceleration in lipolysis (primarily in the femoral-gluteal region), release of fatty acids for hepatic re-esterification leading to hypertriglyceridemia, along with decreased clearance of chylomicron triglyceride[43]. The greater availability of fatty acids increases uptake by the visceral adipocytes, which have a higher lipid turnover rate than peripheral adipocytes, favoring greater net deposition of fat and the development of central adiposity. The underlying causal factor may be the HAART agents or proteins expressed by the virus itself. The net result is increased triglyceride deposition in the liver, central fat, and skeletal muscle; and an increase in proatherogenic lipoproteins or "systemic steatosis".

In this "systemic steatosis" model[44], increased uptake of fatty acids in the liver promotes the synthesis of triglycerides and apolipoprotein B, reduces degradation of apolipoprotein B, and leads to hypertriglyceridemia due to increased production of VLDL. Lipid uptake within the central fat depots is higher than in peripheral fat depots (femoral-gluteal regions). High fat diets cause overexpression of the endothelial cell enzyme lipoprotein lipase, which hydrolyzes lipids in lipoproteins. The differential lipid uptake in the "systemic steatosis" model may therefore be due to an increased sensitivity to lipoprotein lipase-activating hormones (e.g. cortisol in omental adipocytes), thus sequestering fatty acids as di- and tri-glycerides in abdominal visceral depots[44].

### Implications of Fatty Acid Blockade

Inhibition of peripheral lipolysis improves insulin sensitivity in protease inhibitor-treated men with signs of lipodystrophy[45]. On the assumption that increased circulating fatty acids contribute to hepatic insulin resistance and decreased insulin signaling through insulin receptor substrate, Hadigan and colleagues investigated the effects of acute lipolytic blockade with the nicotinic acid analog acipimox, which inhibits fatty acid release[41]. Patients in this study had significant central adiposity (body mass index 28.8 ± 1.9 kg/m^2^; extremity fat 15.9 ± 2.4%; trunk fat 25.8 ± 2.2%; VAT 156.3 ± 2.8 cm^2^; waist-to-hip ratio [WHR] 0.99 ± 0.01). Six of the seven subjects who received acipimox showed improvement in the insulin sensitivity index; and fatty acid area under the curve correlated inversely with insulin sensitivity (r = -0.75, P < 0.05) [41]. More recently, Hadigan et al. reported on the 3-month use of acipimox in HIV-infected men and women with hypertriglyceridemia. Acipimox led to a significant sustained reduction in FFA, decreased rates of lipolysis, and a 34 mg/dL mean reduction in triglyceride concentration and improved insulin sensitivity at 3 months[46]. This supports the concept that excessive release of fatty acids contributes to hepatic adiposity and systemic insulin resistance[47].

### A Consequence of Immune Reconstitution

HIV causes immune dysregulation and immune deficiency; either or both may play a role in fat redistribution. It could be due to the partial immune reconstitution that occurs from successful therapy, or there may be an abnormal immune response to therapy. When CD4 levels start to return to normal after effective HIV therapy, the immune system is not normalized; some patients still experience an opportunistic illness[48]. It is possible that perhaps an immune system continues to be "turned on", to respond to HIV that is no longer detectable, and attacks and kills subcutaneous fat cells–immune reconstitution syndrome. The apoptotic subcutaneous adipose cells would no longer serve as a viable storage depot for triglycerides and FFAs. The atrophic SAT may also release FFAs, which could find their way to VAT. Mitochondrial toxicity due to antiretroviral medication or the HIV itself could result in fat apoptosis. Many potential explanations are being offered, but research has yet to fully elucidate this phenomenon. Though HAART normally induces immune reconstitution, a problem can arise when the immune reconstitution is excessive or inappropriate.

## The GH-Adipocyte Axis

HARS may be considered a state of functional GH deficiency. Normally, GH is released in pulsatile fashion by the somatotrophic cells of the anterior pituitary in response to growth hormone-releasing hormone (GHRH), somatostatin, insulin-like growth factor (IGF-I), thyroid hormone, and glucocorticoids. Although GHRH is released primarily by the hypothalamus, it is also synthesized in the placenta, ovary, testis, lymphocyte, pancreas, and gastrointestinal tract. The complexity of GH regulation suggests the corresponding complexity of its function in the body. GH concentrations vary inversely with excess weight (obesity). Both reduced GH secretion[49,50] and increased clearance have been associated with visceral adiposity in non-HIV patients[51].

Rietschel and colleagues have found normal concentrations of IGF-I but reduced GH concentrations in patients with HARS, suggesting greater receptor sensitivity to GH so that a smaller amount of GH stimulates a normal amount of IGF-I release[52]. Patients in this study were HIV-infected men in whom weight and total body fat were normal but visceral fat depots were enlarged while peripheral fat depots were reduced. The study found 33%-38% prevalence of deficient GH response in lipodystrophic subjects (peak GH stimulatory cut-off values 3.0 μg/mL to 5.0 μg/mL with arginine testing). Reduced mean overnight GH concentration (i.e., reduced basal GH concentration and reduced GH pulse amplitude associated with normal IGF-I) was observed in HAART-treated adults with excess VAT. VAT was the most significant predictor of GH secretion in these subjects.

The actions of the hypothalamic hormone, somatostatin, play a role in GH regulation as well as adipose metabolism. Release of somatostatin is counter regulatory in that it inhibits GH secretion. Koutkia and colleagues compared the difference in GH release by GHRH+arginine stimulation with GHRH alone in 13 HIV-infected men with lipodystrophy and 10 HIV-infected men without lipodystrophy after an overnight fast[27]. VAT in the lipodystrophy group was 197 ± 19 cm^2 ^vs 66 ± 10 cm^2 ^for the non-lipodystrophy men, and 94 ± 13 cm^2 ^for the comparison group. The data demonstrated GH deficiency in 18% of the lipodystrophy group vs 5.9% of the non-lipodystrophy group and 0% of the comparison group, using the stringent criterion of 3.3 ng/ml for peak GH response to GHRH-arginine. Among the lipodystrophy patients, the peak GH response to GHRH-arginine was significantly predicted by VAT (P = 0.008), FFA (P = 0.04) and insulin level (P = 0.007) in regression modeling. These data demonstrate increased frequency of GH deficiency in HIV lipodystrophy patients in conjunction with increased VAT[53]. The addition of arginine to GHRH in the lipodystrophy patients led to a 247% greater increase in GH secretion compared to the lipodystrophy patients who received only GHRH. Since arginine blocks somatostatin's inhibition of GH release, this suggests that patients with HIV lipodystrophy and increased visceral fat have elevated somatostatin tone.

Circulating FFAs may impair GH secretion; conversely, GH replacement demonstrates marked reductions in total and net lipolysis and the availability of FFAs for hepatic reesterification[39]. These results highlight the complex relationship between FFA and GH.

### GH Secretion: Effects of Gender, Race and Fat Distribution

Koutkia et al. demonstrated that HIV-infected men with fat redistribution have significantly lower GH peak responses and higher failure rates to standardized GH stimulation testing in comparison to healthy male control subjects and to HIV-infected women of similar age and body mass[27]. Furthermore, their data suggest that relative GH deficiency is very common among HIV-infected men, especially in those with elevated WHR, even if it was increased due to primary fat loss from the hip region. There was a gender effect – fewer HIV-infected women failed GHRH + arginine stimulation. Among men, a cutoff of 7.5 ng/ml for peak GH was used to show a failure rate of 37% vs 8% for control groups (P = 0.004). Thus, one-third of the men with fat redistribution in this study can be considered at least relatively GH deficient. In contrast to patients with true GH deficiency, e.g. due to a pituitary tumor or radiation, patients in this study were presumed to have otherwise normal pituitary function. Among women, no specific cutoff could be determined to separate HIV-infected and control subjects. This may be due to the effects of estrogen on the GH/IGF-1 axis in this relatively young population and might be different for an older or postmenopausal population.

In the same study, there were differences by race in these patients with HIV and fat redistribution. Non-Caucasian HIV-infected men had higher GH responses to stimulation than Caucasian male HIV-infected subjects. In contrast, non-Caucasian HIV-infected women, compared to Caucasian HIV-infected women, had lower GH responses.

## Conclusion

The underlying pathophysiologic mechanisms responsible for HARS are multifactorial and not well understood at present. These may include dysfunction of the GH axis, alterations in immune function, along with increased levels of insulin and various other hormones, cytokines and proteins. Factors contributing to the dysfunction of the GH axis may also be multifactorial. Further understanding of these pathophysiological factors is needed for a complete understanding of HIV lipodystrophy. We can expect that future research will gradually elucidate the complex pathogenic mechanisms and the disfiguring, as well as health-threatening, conditions associated with HARS.

## Abbreviations

11-β-HSD1 11-beta hydroxysteroid dehydrogenase type 1

AIDS Acquired immunodeficiency syndrome

FFA Free fatty acid

FRAM Fat Redistribution and Metabolic Changes in HIV Infection

GH Growth hormone

GHRH Growth hormone releasing hormone

HAART Highly active antiretroviral therapy

HARS HIV-associated adipose redistribution syndrome

HIV Human immunodeficiency virus

IGF-1 Insulin-like growth factor-1

IL Interleukin

PAI-1 Plasminogen activator inhibitor type 1

SAT Subcutaneous adipose tissue

TNF-α Tumor necrosis factor-α

VAT Visceral adipose tissue

VLDL Very low density lipoprotein

WHR Waist:hip ratio

## Competing interests

Dr. Eric Freedland is currently an employee of EMD Serono, Inc. EMD Serono, Inc. holds rights to Serostim, a brand of recombinant human growth hormone, which has been submitted to the FDA for approval for the treatment of HARS. Dr. Balasubramanyam has received honorarium for a consultative meeting with EMD Serono regarding the possible use of growth hormone to treat HIV HARS. The present manuscript does not discuss growth hormone treatment of HIV HARS, but it does discuss growth hormone deficiency in this condition. Dr. Lichtenstein has nothing to disclose. Dr. Sekhar has received honoraria for consultative meetings with EMD Serono regarding the possible use of growth hormone to treat HIV lipodystrophy. The present manuscript does not discuss growth hormone treatment of HIV lipodystrophy, but it does discuss growth hormone deficiency in this condition.

## Authors' contributions

All authors were involved in drafting the manuscript and provided extensive comments and review. All authors performed analysis and interpretation of data. All authors have read and approved the final manuscript.
